# The Effectiveness of Technology-Based Strategies to Promote Engagement With Digital Interventions: A Systematic Review Protocol

**DOI:** 10.2196/resprot.3990

**Published:** 2015-04-28

**Authors:** Ghadah Alkhaldi, Fiona L Hamilton, Rosa Lau, Rosie Webster, Susan Michie, Elizabeth Murray

**Affiliations:** ^1^E-Health UnitResearch Department of Primary Care and Population HealthUniversity College LondonLondonUnited Kingdom; ^2^Research Department of Clinical, Educational and Health PsychologyUniversity College LondonLondonUnited Kingdom

**Keywords:** systematic review, Internet, computers, Web-based interventions, engagement, adherence, attrition, usage

## Abstract

**Background:**

Digital interventions provide effective and potentially cost-effective models for improving health outcomes as they deliver health information and services that are widely disseminated, confidential, and can be tailored to needs of the individual user. Digital interventions have been used successfully for health promotion, mental health, and for enabling self-management of long-term conditions. However, their effectiveness is limited by low usage rates, with non-engagement a major challenge. Hence, it is crucial to find effective strategies to increase user engagement with digital interventions.

**Objective:**

This systematic review will aim to evaluate the effectiveness of technology-based strategies to promote engagement with digital interventions.

**Methods:**

We will follow Cochrane Collaboration guidelines on systematic review methodology. The search strategy will be executed across seven e-databases (including MEDLINE, EMBASE, PsycINFO, CINAHL) using the concepts “digital intervention” and “engagement”, limited by study type (randomized controlled trial). Grey literature and reference lists of included studies will be searched. Titles and abstracts will be independently screened by 2 authors. Then the full text of potentially eligible papers will be obtained and double screened. Data from eligible papers will be extracted by 1 author and checked for accuracy by another author. Bias will be assessed using the Cochrane bias assessment tool. Narrative synthesis will report on all included studies, and where appropriate, data will be pooled using meta-analysis. All findings will be reported according to the Preferred Reporting Items for Systematic Reviews and Meta-Analyses guidelines. Sources of heterogeneity will be further investigated if required.

**Results:**

Our research is in progress. The final draft of the systematic review is being written and will be submitted before the end of 2015.

**Conclusions:**

The review findings will inform researchers and digital intervention providers about optimal use of technology-based strategies to promote engagement with digital interventions.

**Trial Registration:**

PROSPERO International Prospective Register of Systematic Reviews: CRD42014010164; http://www.crd.york.ac.uk/PROSPERO/display_record.asp?ID=CRD42014010164#.VTZmmiFViko (Archived by WebCite at http://www.webcitation.org/6XxQC8fT8).

## Introduction

### Digital Interventions

Digital interventions (DIs) are programs that provide information and support (emotional, decisional, and/or behavioral) for physical and/or mental health problems via a digital platform (ie, website, computer) [1]. DIs have been developed and used for numerous health issues including improving self-management of long-term conditions [2] (eg, diabetes [3] and asthma [4]), health promotion for sexual health [1], reducing excessive alcohol consumption [5-7], smoking cessation [8,9], increasing physical activity [10,11], and mental illness (eg, depression [12]). DIs can potentially provide a convenient gateway for patients to access and receive tailored and private health information and services [1,5]. Numerous systematic reviews have confirmed the potential effectiveness of DIs in improving health behaviors and health outcomes [1-5,7-14]. However, overall, effect sizes tend to be small and many reviews have noted substantial heterogeneity. A common problem for DIs is lack of engagement, or attrition from the intervention [15].

### Engagement With Digital Interventions

Research suggests that the effectiveness of a DI can be mediated by the user’s level of engagement, and there appears to be a dose-response relationship [13,16-18]. For example, one randomized controlled trial (RCT) found that users of a smoking cessation DI had better quit outcomes if they had a higher number of logins (OR 1.19, 95% CI 1.08-1.31) [19]. In another RCT of an intervention to increase vegetable and fruit intake, there was a positive association between usage of the intervention and increased intake of fruit and vegetables [20]. Further, a descriptive systematic review exploring the relationship between engagement and DI outcomes found a positive association between engagement with the intervention and outcomes for interventions targeting physical health [21]. Although this association could be due to reverse causality, where users who make most change (for other reasons) ascribe this change to the DI and hence engage with it, it is not unreasonable to suggest that non-use or suboptimal use of a DI is likely to limit its effectiveness [15,22]. Hence, there is considerable interest in methods of improving user engagement with DIs.

### The Use of Prompts to Engage Digital Intervention Users

One potential strategy for improving engagement that has been explored is the use of prompts or reminders [15,23,24]. An early meta-synthesis of DIs for behavior change found that use of text messages, phone calls, and email prompts had a significant enhancing effect on behavior change: effect size (d+)=0.81, CI 0.14-1.49; 0.35, CI 0.09-0.61; and 0.18, CI 0.07-0.29, respectively [25]. A systematic review also found that periodic email and phone prompts used for behavior change were effective compared to control groups either receiving non-technological prompts or no prompts [26]. However, neither of these reviews focused primarily on promoting engagement with the intervention. Both had a primary aim of determining the overall effectiveness of digital interventions for behavior change. Brouwer et al (2011) undertook a review of literature published between 1995 and 2009 to explore which strategies have been integrated into interventions to improve engagement, and what the relative effectiveness of these strategies were. This review found considerable heterogeneity but suggested that regular contacts by email or phone appeared to result in greater number of logins [27]. Human contact (eg, regular phone calls) may considerably add to the cost of delivery of digital interventions and may therefore undermine one of the potential benefits of digital interventions, namely the low marginal cost per additional user [28].

To our knowledge, there have been no reviews focusing specifically on automated or technological methods of promoting engagement with digital interventions. This review addresses this gap.

### Aim and Objectives

Our overall aim is to evaluate the effectiveness of technology-based strategies to promote engagement with DIs.

Specific objectives are (1) to describe technology-based strategies to promote engagement with DIs, (2) to assess the effectiveness of technology-based strategies in promoting engagement with digital interventions, (3) to explore whether different characteristics such as timing, frequency, duration, content, sender, mode of delivery, or use of theory are associated with differential effectiveness, and (4) describe the cost of technology-based strategies to promote engagement with digital interventions.

## Methods

### Design

This study is a systematic review of RCTs and quasi-RCTs following Cochrane methodological guidance [29]. A structured approach has been used to build the eligibility criteria, using PICOS (Participants, interventions, comparisons, outcomes and study designs) [30].

### Definitions

The systematic review was designed to be comprehensive and inclusive, thus the following definitions were used:

- Digital interventions are programs that provide information and support (emotional, decisional, and/or behavioral) for physical and/or mental health problems via a digital platform specifically a website or a computer [1]. The definition was chosen because it includes offline and online interventions and specifies the purpose of the DI without limiting it by listing specific characteristics [31].
- Engagement has been defined in the literature by its outcome measures such as the number of logins/visits, number of modules used, duration of time spent on DI or number, and type of pages viewed and visited [17,32,33]. This way of defining engagement usually depends on the characteristics of the DI; for example, if the DI consists of modules, then engagement will be defined by the number of completed modules. In addition, engagement has been categorized into three phases: (1) visiting the DI for the first time, (2) prolonging the first visit, and (3) revisiting the DI [34], which depends to some extent on the goal of the DI and whether it has to be used once or repeatedly. In this systematic review, the third phase of engagement will be targeted, the user’s regular interaction with part or all of the DI. The most appropriate measures for this definition are the number of participants who visited the DI (logged-in to the website) and/or the number of visits/logins, as they bridge the gap between the engagement strategy and users interacting or accessing the website [27,35], but other measures will be considered depending on the included papers.
- It is important to differentiate between disengagement from a DI (non-usage attrition), and disengagement from an online trial of a DI, that is, loss to follow-up (dropout attrition) [15]. For example, one study of a DI for workplace health promotion reported higher non-usage attrition in controls compared with intervention participants (who received regular emails) but higher dropout attrition in the intervention group than the control group [36]. Similarly, another study examined the relationship between dropout attrition and disengagement from a DI and found that the relationship between these two is complex and that factors associated with greater adherence to a trial or better engagement to a DI were not similar [23].
- Based on the definition of engagement above, technology-based engagement promoting strategies will be defined as digital and analogue technology methods used to promote the user’s regular interaction with all or part of the DI, including but not limited to landline phone calls, cell phone calls, text messages, multimedia messages, emails, automated voice calls, or faxes. Examples of interventions that will be included are a computerized treatment program with cell phone text messages that remind the user to visit the program or a blood pressure self-monitoring website that sends email prompts to users to enter their pressure readings on the website.

### Data Sources and Search Methods

A comprehensive search strategy has been developed to ensure we identify all potentially relevant studies. The strategy was developed by the lead author together with an information specialist and reviewed by the entire team. The strategy was informed by previous search strategies for reviews of DIs available in the literature. It combined the two concepts of digital interventions and engagement, limited by study type (RCT).

Hand searching was done to pilot the electronic database search strategy. Issues from last 2 years (2012-2013) of the *Journal of Medical Internet Research (JMIR)* were searched to find related articles and test whether the articles were identified and the search strategy was adjusted accordingly. The validity of the search strategy was also assessed by taking seven known RCTs of technological strategies to promote engagement with DIs and checking to see if they were identified in a MEDLINE search using the strategy (see Multimedia Appendix 1).

The Medline thesaurus Medical Subject Headings (MESH) terms were refined for each database, and unpublished data will be sought in the form of conference proceedings (Conference Proceedings Citation Index, formerly ISI Proceedings). References of the included studies and issues of key journals such as JMIR will be hand searched, and any papers citing included or key papers will also be screened.

The following databases will be searched from inception with no language restrictions: the Cochrane Central Register of Controlled Trials (CENTRAL); General international health care electronic bibliographic databases: MEDLINE and EMBASE; and social science, education, psychology and nursing electronic bibliographic databases: ISI Web of Science, Education Resources Information Center (ERIC), PsycINFO, and Cumulative Index to Nursing and Allied Health Literature (CINAHL).

### Articles Screening and Selection

**Figure 1:**
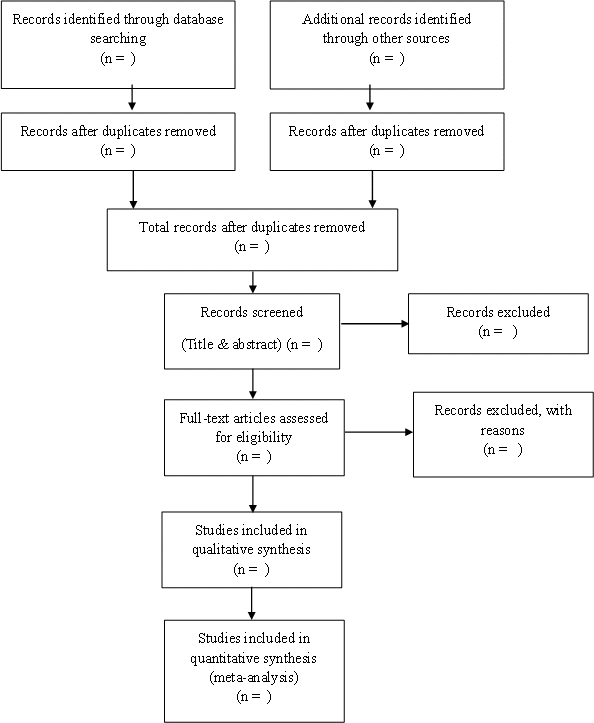
PRISMA flow diagram.

All citations identified by the search strategy will be downloaded to the reference manager EndNote X5 and de-duplicated. Studies will be independently double screened. Full text manuscripts for potentially eligible articles will be obtained, and authors will be contacted directly for articles that were not retrievable through library sources. The full text articles will be assessed for eligibility by 2 authors (GA and EM). Any disagreement will be resolved by discussion with reference to the inclusion and exclusion criteria or if necessary with input from a third reviewer (FH). Justification for exclusion will be recorded, and a Preferred Reporting Items for Systematic Reviews and Meta-Analyses (PRISMA) flowchart will be constructed to show search, screening, and selection results (see Figure 1).

### Inclusion Criteria

#### Participants

Participants will be all adults, aged 18 years old or over. There will be no limitations on gender, socioeconomic status, ethnicity, or health status. Participants may use the intervention in any setting.

#### Interventions

The intervention of interest is technology-based strategies to promote engagement with digital interventions. The interventions have to meet the definition of the strategies described above.

#### Comparisons

We will include three groups of comparators: (1) minimal or inactive comparators, such as no strategy, (2) non-technological strategies such as printed materials or face-to-face contact, and (3) alternative technology-based strategies (eg, where the effects of email prompts are compared to the effects of text-message prompts). This third comparator will be used to explore relative effectiveness of the different strategies.

#### Outcomes

##### Primary Outcomes

The primary outcome will be engagement with the DI, which may be recorded as the number of logins/visits, number of pages visited, number of sessions completed, time spent on the intervention website, and number of DI components/features used. To simplify interpretation of the outcome measures, they will be categorized into dichotomous and continuous engagement outcomes, for example:

- Dichotomous engagement outcome: any dichotomous measure of how participants engaged with the DI such as proportion of participants who visited the DI, or proportion of participants who completed a pre-specified number of modules.
- Continuous engagement outcome: any continuous measure of how participants engaged with the DI such as number of visits or page views.

##### Secondary Outcomes

Two types of secondary outcomes will be selected: (1) adverse outcomes such as users feeling frustrated and bothered by engagement prompts, users experiencing a loss of self-esteem due to not being able to engage with the DI, users receiving prompts with wrong information or links to the DI, and exclusion for users who are not able to receive the engagement prompt, and (2) economic outcomes, which are costs associated with strategies promoting engagement to inform future cost-effectiveness analysis. All outcomes measured in the studies that meet our inclusion criteria will be included whether they are objective or self-reported.

##### Study Designs

Studies of RCTs or quasi-RCTs will be included. Trials can either be trials of DIs that used strategies promoting engagement or they can be trials evaluating strategies specifically. Economic evaluation will be included if they were conducted alongside the main trial.

### Exclusion Criteria

Exclusion criteria will comprise the following: (1) interventions targeted exclusively at health professionals (eg, computer-based decision aids to assist health professionals in making decisions with regards to treatments), (2) trials where attrition from trial and attrition from intervention are non-distinguishable, and (3) trials where the effect of the DI components cannot be separated from the effect of the engagement promoting strategy; for example, when trials where the DI is not compared to another DI (eg, a website to lose weight with email prompts compared with dietitian face-to-face sessions with emails from the dietitian) or when a DI with an engagement strategy is compared to a different DI without engagement strategy (eg, an enhanced version of the DI with email prompts compared to a basic version without engagement prompts).

### Data Abstraction

Data will be extracted using an adapted version of the Cochrane Consumers and Communication Review Group data extraction template. The data extraction form will be piloted and changes will be documented. Standard information will be collected including study references, design, aims and objectives, funders, setting, health condition/health behavior, population details, exclusion and inclusion criteria, digital intervention, analytical methods, follow-up duration and rates, results, and risk of bias. In addition, we will extract full details of the engagement strategy, including timing, frequency, duration, content, sender, mode of delivery (eg, email, text message), and use of theory. We will apply a taxonomy of behavior change techniques (BCT) developed for use with digital interventions [37] to describe and codify the content of the engagement strategies. Data will be extracted from the included studies by 1 review author (GA), and a second review author (FH) will independently verify the extracted data. Application of the BCT taxonomy will be undertaken by the lead author (GA) and checked by a second author with experience using this taxonomy (RW).

Disagreement will be resolved by discussion between the 2 authors. If no agreement can be reached, a third author (EM) will decide and reasons for the decision documented. If any information is missing or needs to be clarified, authors will be contacted.

### Data Analysis and Synthesis

#### Measurement of Treatment Effect

The appropriate effect measure will be determined depending on the type of data. For the primary outcome, website metrics will either be continuous or dichotomous. For dichotomous outcomes, odds ratio or relative risk and their 95% confidence intervals will be used. For continuous outcomes, mean difference with 95% confidence intervals or standardized mean difference will be used.

#### Unit of Analysis Issues

It is anticipated that most studies will have randomized DI users to either intervention or control groups, therefore the unit of analysis will be the individual.

#### Dealing With Missing Data

As primary outcomes measures (ie, website metrics) are automatically generated during a DI, it is anticipated that missing data will most likely be in secondary outcomes. Where missing data present a clear bias to the study outcomes, it will be noted and discussed with the research team and the authors will be contacted directly for clarification. Where the risk of bias cannot be mitigated, studies will be included only in the narrative part of the systematic review.

#### Data Analysis

Results will be reported according to the PRISMA guidelines [30]. Data from included studies will be tabulated to allow for narrative description of the results. This also allows for assessment of heterogeneity in terms of participants, DI and strategy, outcomes, comparator, study design, and quality of studies (risk of bias). Where appropriate, data will be summarized statistically by meta-analysis according to Cochrane systematic review methodology. Data will be pooled using fixed effects and random effects model. The results will be presented for three comparator types: minimal or inactive comparators, non-technological strategies, and alternative technology-based strategies.

Where possible, we will use the number of participants who visited the DI (logged-in to the website) or the number of visits/logins, as these are the most appropriate indicators for engagement [27,35]. The longest follow-up period available will be chosen, as it is important to demonstrate sustained change.

Due to the variable nature of the interventions, heterogeneity is expected and it will be assessed using the I^2^ statistic to quantify the amount of variation in results across studies beyond that expected from chance [30]. Sensitivity analysis will be conducted according to the Cochrane handbook recommendation by excluding trials with poor quality to determine their effects on the meta-analysis. Reporting bias will be assessed through visual inspection of funnel plots.

Data on characteristics of engagement strategies, and adverse and economic outcomes will be described narratively and summarized statistically if possible.

### Critical Appraisal Techniques

An assessment of risk of bias will be done based on the Cochrane risk of bias assessment tool [29]. The following criteria will be used:

- Was the allocation sequence adequately generated?
- Was allocation adequately concealed?
- Was knowledge of the allocated interventions adequately prevented during the study (blinding)?
- Were incomplete outcome data adequately addressed?
- Are reports of the study free of suggestion of selective outcome reporting?
- Was the study free of other problems that could put it at a risk of bias? Including but not limited to differences in baseline characteristics between groups, validity and reliability of outcome measures, sample size, and power.

Studies will be categorized as low risk of bias, high, or unclear. A risk of bias graph and summary table will be generated. The bias assessment will be done by 1 author (GA) and will be checked by another author (FH). Any discrepancies will be resolved by a third author (EM).

### Consumer Participation

If possible, developers, researchers, individuals, or groups interested in the review will be asked whether the protocol addresses priorities and if they can help in interpretation of data synthesis and to inform the discussion and conclusion of the systematic review.

## Results

Our research is in progress. The final draft of the systematic review is being written and will be submitted before the end of 2015.

## Discussion

This review will present an unbiased and detailed summary of the current and available evidence regarding technological strategies that promote engagement with DIs. Results of this review will enable researchers and DI providers to make optimal use of technological prompts to enhance engagement with DIs.

## Multimedia Appendix 1

MEDLINE search strategy.

## Abbreviations

DI: digital intervention
PRISMA: Preferred Reporting Items for Systematic Reviews and Meta-Analyses
RCT: randomized controlled trial
