# Supplementary material for: The Effectiveness of Technology-Based Strategies to Promote Engagement With Digital Interventions: A Systematic Review Protocol
Source: JMIR Res Protoc. 2015 Apr 28;4(2):e47. doi: 10.2196/resprot.3990 (PMC4429223; doi:10.2196/resprot.3990)
Supplement: Supplementary file 1 [file resprot_v4i2e47_app1.pdf]

MEDLINE

1 Computer Communication Networks/

2 Medical Informatics/

3 Medical Informatics Applications/

4 Decision Support Techniques/

5 Educational Technology/

6 Audiovisual Aids/

7 Telecommunications/

8 Public Health Informatics/

9 Computer-Assisted Instruction/

10 Hypermedia/

11 Internet/

12 reminder system/

13 exp Telemedicine/

14 computer literacy/

15 exp Telephone/

16 exp Computers, Handheld/

17 exp Hotlines/

18 (Internet or local area network$).ti,ab.

19 (Decision tree$ or decision aid$).ti,ab.

20 (Software or software design).ti,ab.

21 (CD-ROM or Compact disk$ or cd-rom or CDROM).ti,ab.

22 (Electronic mail$ or e-mail$ or email$).ti,ab.

23 (World wide web or world-wide-web or www or world-wide web or worldwide web or website$).ti,ab.

24 (Video recording or video record$ or DVD).ti,ab.

25 (Online or on-line).ti,ab.

26 (Chat room$ or chatroom$).ti,ab.

27 (blog$ or web-log$ or weblog$).ti,ab.

28 (bulletin board$ or bulletinboard$ or messageboard$ or message board$).ti,ab.

29 Interactive health communicat$.ti,ab.

30 interactive televis$.ti,ab.

31 interactive video$.ti,ab.

32 Interactive technology.ti,ab.

33 Interactive multimedia.ti,ab.

34 (E-health or ehealth or electronic health).ti,ab.

35 Consumer health informatic$.ti,ab.

36 Virtual reality.ti,ab.

37 (surf$ adj4 web$).ti,ab.

38 (surf$ adj3 internet).ti,ab.

39 (text message$ or short message$ service$ or SMS).ti,ab.

40 (multimedia message$ or multimedia message$ service$ or MMS).ti,ab.

41 (voicemail$ or voice mail$ or interactive voice response system$).ti,ab.

42 social network$.ti,ab.

43 (m-health or mobile health or mobilehealth or mhealth).ti,ab.

44 (tele-care or telecommunication care or telecare).ti,ab.

45 (tele-health or telecommunication health or telehealth).ti,ab.

46 (phone$ or telephone$ or tele-phone$ or smartphone$ or smart-phone$).ti,ab.

47 (cyberpsychology or cybertherap$ or etherap$ or ecounsel$).ti,ab.

48 (Electronic message$ or e-message$ or emessage$).ti,ab.

49 (voice adj (response or recognition or messag$ or mail$ or service$ or system$)).ti,ab.

50 ((prerecorded or pre-recorded) and (voice$ or hotline$ or hot line$ or call$ or messag$)).ti,ab.

51 (computer$ adj3 (pocket$ or palm$ or hand-held or handheld)).ti,ab.

52 (personal adj digital adj assistant).ti,ab.

53 (digital intervention$ or mobile device$).ti,ab.

54 (Computer$ or microcomputer$).ti,ab.

55 or/1-54

56 randomized controlled trial.pt.

57 controlled clinical trial.pt.

58 randomized.ab.

59 placebo.ab.

60 clinical trials as topic.sh.

61 randomly.ab.

62 trial$.ti,ab.

63 or/56-62

64 exp animals/ not humans.sh.

65 63 not 64

66 Patient Compliance/

67 Patient Dropouts/

68 exp consumer participation/

69 Guideline Adherence/

70 ((patient$ or user$ or consumer$ or client$ or participant$ or people) adj5 (attrition or participat$ or adher$ or engage$ or feedback or remind$ or prompt$ or invit$ or educat$ or interact$ or retention or retain)).ti,ab.

71 or/66-70

72 55 and 65 and 71
